# Multimodality Treatment of Pulmonary Sarcomatoid Carcinoma: A Review of Current State of Art

**DOI:** 10.1155/2022/8541157

**Published:** 2022-03-25

**Authors:** Lin Zhang, Weihao Lin, Zhenlin Yang, Renda Li, Yibo Gao, Jie He

**Affiliations:** ^1^Department of Oncology, Renmin Hospital of Wuhan University, Wuhan, China; ^2^Department of Thoracic Surgery, National Cancer Center/National Clinical Research Center for Cancer/Cancer Hospital, Chinese Academy of Medical Sciences and Peking Union Medical College, Beijing, China; ^3^State Key Laboratory of Molecular Oncology, National Cancer Center/National Clinical Research Center for Cancer/Cancer Hospital, Chinese Academy of Medical Sciences and Peking Union Medical College, Beijing, China

## Abstract

Pulmonary sarcomatoid carcinoma (PSC) is an unconventional non-small-cell lung cancer (NSCLC) that is currently managed under guidelines used for conventional NSCLC and has poor survival. Surgery is the optimal choice for resectable PSC, and the prevalence of mutations in this type of tumor laid the foundation for novel systemic therapies such as targeted therapy and immunotherapy. PSC is resistant to chemotherapy and radiotherapy, and the effects of the 2 therapies are controversial. Targeted therapies have been reported to confer survival benefits, and savolitinib, an oral selective MET tyrosine-kinase inhibitor, has been approved in metastatic patients with MET exon 14 skipping mutations. Expression and positive rate of programmed death ligand 1 in PSC are high; our previous research has also revealed a high mutational burden and a T-cell-inflamed microenvironment of PSC. Correspondingly, immune checkpoint inhibitors have shown preliminary antitumor effects (overall response rates of 40.5% (15/37) and 31.6% (6/19) in two retrospective studies, respectively) in PSC patients. In summary, patients should receive operations at an early stage and multimodality treatments are needed to maximize the benefits of patients with advanced disease.

## 1. Introduction

**Figure 1:**
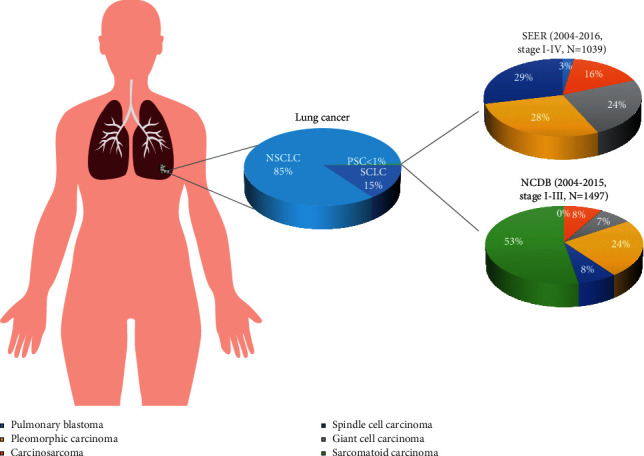
Proportions of pulmonary sarcomatoid carcinoma in lung cancer and different subtypes of pulmonary sarcomatoid carcinomas. Data of SEER were extracted from the research by Gang et al. (PMID: 33569423), and those of NCDB were from the research by Abdallah et al. (PMID: 33678508). NSCLC, non-small-cell lung cancer; SCLC, small-cell lung cancer; PSC, pulmonary sarcomatoid carcinoma; SEER, Surveillance, Epidemiology, and End Results database; NCDB, National Cancer Database.

Pulmonary sarcomatoid carcinoma (PSC) is a group of rare types of non-small-cell lung cancer (NSCLC) and is composed of 5 different pathologic types: carcinosarcoma, spindle cell carcinoma, pleomorphic carcinoma, giant cell carcinoma, and pulmonary blastoma (Figure 1) [1]. The proportion of PSC among all lung malignancies is low [2]. According to analyses of the National Cancer Database, the proportion was less than 1% [3, 4]. In the Surveillance, Epidemiology, and End Results (SEER) database, the proportion was 0.4% (3647/878810) [5]. Meanwhile, the incidence of PSC among the population has slowly decreased from 0.120/100,000 in 2004 to 0.092/100,000 in 2015 [6].

PSC was more common in aged male patients with a smoking history [2, 6, 7]. The prognosis of PSC is poorer than other forms of NSCLC stage by stage [5, 6]. For PSC, the survival rates at 1, 3, and 5 years were 33.7%, 18.4%, and 14.4%, respectively; the median OS for stage I-II disease was 16.9 months, that for stage III disease was 5.8 months, and that for stage IV disease was 5.4 months [8]. The patients with pleomorphic carcinoma had a much worse prognosis [2]. Up to now, there have not been any phase 3 randomized controlled trials exploring systemic therapies for advanced PSC yet, while trials of other phases are also scarce [9, 10]. The standard treatment strategies for PSC are still under debate, and effects of chemotherapy and radiotherapy are controversial. Therefore, it is necessary to summarize the current knowledge about different therapies for this rare disease and provide multimodality treatments for patients to maximize their clinical benefits.

## 2. Surgical Resection

Surgical resection is recommended as the optimal treatment for early-stage NSCLC, and considerable research has also demonstrated that surgery promotes the survival of PSC patients, especially in the initial stages of the disease [11, 12]. Consistent with this, surgery remained to be an independent favorable prognostic factor for survival in retrospective analyses [2, 13, 14]. The conclusion has been demonstrated in analyses using the cohort from the SEER database and other independent cohorts.

Gang et al. reviewed PSC patients from the SEER database between 2004 and 2016 and collected 1039 eligible cases; surgery was confirmed to be an independent favorable prognostic factor, with a hazard ratio (HR) of 0.484 (*P* < 0.001), as revealed by multivariate analyses [15]. Significant association of surgery with OS was also identified by multivariate analyses performed by Sun et al. using 1640 PSC patients collected from the SEER database (from 1988 to 2014, HR = 0.44, *P* < 0.01) and by Chen et al. using 1049 PSC patients collected from the SEER database (from 2004 to 2015, HR = 0.40, *P* < 0.001) [6, 16].

Zeng et al. retrospectively collected information of 262 consecutive PSC patients in our center and found that patients undergoing surgical treatment had a significantly better median survival time compared to those receiving nonsurgical treatment (11.0 versus 23.0 months, *P*=0.016); they concluded that surgery should be the mainstay treatment for operable cases [13]. Lin et al. reviewed 69 PSC patients who received treatment in Sun Yat-sen University Cancer Center, an institution in southern China; multivariate Cox regression analyses revealed that complete surgical resection was one of the independent prognostic factors and those who received complete resection had significantly better overall survival (OS) than those who did not (relative risk 2.590, *P*=0.032) [2]. In a cohort from Shanghai Pulmonary Hospital, Sun et al. identified surgery as one of the prognostic factors for OS (HR = 0.51, *P*=0.02) and progression-free survival (PFS) (HR = 0.46, *P* < 0.01) through multivariate analyses [16].

## 3. Chemotherapy

Although surgery is a preferred treatment option, it is not viable for patients who are diagnosed in an advanced stage. Alternatively, platinum-based doublet chemotherapy used to be a first-line treatment for unresectable patients without a targetable driver oncogene; platinum-based chemotherapy plans as a neoadjuvant or adjuvant therapy have also been recommended by the National Comprehensive Cancer Network in NSCLC treatment [11, 17]. However, the role of chemotherapy in PSC has been controversial. While some studies showed that chemotherapy is a favorable prognostic factor for OS and that perioperative chemotherapy reduced the risk of recurrence in PSC patients of a specific subgroup, others revealed that neither neoadjuvant nor adjuvant chemotherapy improved patients' survival for those with early-stage disease [2, 18, 19].

### 3.1. Perioperative Chemotherapy

Despite that both neoadjuvant and adjuvant chemotherapy have improved survival in patients with resectable NSCLC [20, 21], the effect of perioperative chemotherapy in PSC is controversial. In the cohort from Mayo Clinic (*n* = 127), while combination of adjuvant or neoadjuvant chemotherapy with surgery is a favorable prognostic factor for survival (*P*=0.04), perioperative chemotherapy did not add extra survival benefit to PSC patients in survival compared to surgery alone (18.6 months versus 14.5 months, *P*=0.23) [7]. In a cohort from Memorial Sloan Kettering between 2000 and 2010, among 12 neoadjuvant chemotherapy patients with evaluable objective responses, neoadjuvant chemotherapy led to minor responses in 4 patients and major responses in 5 patients, whereas the effect of neoadjuvant chemotherapy on patients' survival was not mentioned; in the subset analyses, a recurrence benefit to perioperative chemotherapy in patients with stage Ib-IIa was not identified (*P*=0.82), while the benefit was seen in patients with IIb-IIIa disease (*P*=0.022) [18].

The effect of adjuvant chemotherapy in PSC has been demonstrated in some studies, but there is still debate. In a Chinese cohort by Zeng et al. (*n* = 262), while the median survival time was similar between patients receiving surgery alone and those undergoing surgery with chemotherapy in the entire cohort, the use of surgery followed by adjuvant therapy significantly prolonged median survival time in stage III patients (*P*=0.003) [13]. The study of Shanghai Pulmonary Hospital cohort by Sun et al. (*n* = 175) also exhibited that adjuvant chemotherapy was significantly associated with OS in surgically treated PSC patients (HR = 0.78, *P*=0.03) [16]. Analyses of the SEER database also revealed a survival benefit conferred by adjuvant chemotherapy in surgically treated PSC patients, but the difference was only significant in stage II-III disease after propensity score matching [16, 22]. In contrast, in another 2 cohorts from Mayo Clinic (*n* = 127) and 2 French centers (*n* = 97), adjuvant chemotherapy did not improve survival of patients compared to surgery alone [7, 23]. Interestingly, platinum-based doublet conferred better OS compared to platinum alone (*P* < 0.001) [23]. Thus, the effect of perioperative chemotherapy in PSC needs to be verified in decisive clinical trials.

### 3.2. First-Line/Palliative Chemotherapy

While PSC is resistant to chemotherapy, benefits to patients with advanced PSC have been shown in several studies. Platinum doublet used to be the first-line chemotherapy plan for unresectable NSCLC patients without a targetable driver oncogene [17]. However, in NSCLC patients receiving chemotherapy, sarcomatoid histology type is more common in chemotherapy-refractory patients than in patients with controlled disease (*P*=0.057) [24]. In a retrospective analysis, no significant difference was observed in PFS in patients who have received chemotherapy treatment compared to those who have not; while patients receiving first-line platinum-based chemotherapy was slightly associated with better OS (HR = 0.92, *P*=0.027), the patients also had a better performance status (0-1) than those who did not (93% versus 23%; *P* < 0.0001), which might be a confounding factor [25]. The results implicated that PSC is resistant to chemotherapy. However, there are studies showing more inspiring results. Analyses of the SEER database revealed that chemotherapy is an independent, favorable prognostic factor for OS of PSC patients [15, 16, 22]. The cohort study by Sun et al. also demonstrated that chemotherapy is predictive of OS (HR = 0.38, *P* < 0.01) and PFS (HR = 0.46, *P* < 0.01) in PSC patients [16]. A study of the Mayo cohort revealed that palliative chemotherapy in patients with advanced disease led to tumor responses, with an overall response rate (ORR) of 8.0% and a disease control rate (DCR) of 48.0% among 25 patients [7]. A study by Vieira et al. also revealed that first-line chemotherapy led to a 16.5% ORR and a 47.5% DCR and patients evaluated as disease control at first-line chemotherapy had significantly improved OS (HR = 0.38, *P* < 0.001) [25]. In summary, the effect of first-line/palliative chemotherapy in unresectable PSC is likely but still needs to be verified in integrated analyses.

## 4. Radiation Therapy

Similar to chemotherapy, while radiation therapy was reported to benefit PSC patients, the effect of perioperative radiation therapy is controversial. Radiation therapy has been playing a predominant role in treating early and locally advanced NSCLC [26]. NSCLC patients who have medically inoperable early-stage tumor, refuse surgery, are high-risk surgical candidates, or have stage IV disease should be considered candidates for radiation therapy [11]. The studies using the SEER database concluded that radiation was a predictor of OS (*P*=0.041); more specifically, radiation therapy improved OS of patients with stage I-III disease comparing to those receiving no specific treatment (*P* < 0.001) [15, 27]. Radiotherapy conferred better median and 5-year OS in patients who did not undergo surgery (*P* < 0.001), while neoadjuvant radiotherapy seemed to have conferred more survival benefit than adjuvant radiotherapy (*P*=0.018) [28]. However, the effect of adjuvant radiotherapy remained to be clarified. After propensity score matching, surgery seemed to have led to better 5-year OS than the combination of surgery and radiotherapy (*P*=0.052) [27]. The cohort by Sun et al. (*n* = 175) showed that adjuvant radiation therapy was significantly associated with OS in PSC patients [16]. Research by Maneenil et al. revealed that, while treatment with surgery plus adjuvant chemotherapy/radiotherapy prolonged patients' survival, the difference in the median survival was not statistically significant between the patients treated with surgery alone versus those treated with surgery plus adjuvant therapy [7]. Thus, the effect of adjuvant radiotherapy in different settings needs to be verified in future studies with large sample sizes.

## 5. Mutations and Targeted Therapy

PSC is highly invasive and resistant to traditional systemic therapies. Consequently, identification of actionable molecular targets for such infrequent and aggressive diseases becomes critical for choosing treatment options. Although PSCs are thought to be chemorefractory, a considerable proportion of PSCs harbors potentially targetable genomic alterations [29]. The most frequently mutated genes across different studies include TP53, KRAS, MET, EGFR, BRAF, HER2, and RET [29–33], which are generally consistent with those in our research [34]. In the study by Zhou et al., 91.4% (53/58) of the Chinese patients with pure PSC harbored at least one mutation; 45% of the patients harbored at least one actionable alteration, and TP53 was the most frequent mutation (74.1%, 43/58) [30]. In our cohort, whole exome sequencing on DNA samples collected from 56 PSC patients revealed that 98.2% (55/56) of the patients harbor gene mutations and TP53 was the most frequently mutated gene (78.6%, 44/56), followed by CDKN2A (28.6%, 16/56) and MYC (25.0%, 14/56) [34]. Despite the prevalence of mutation genes, reports of effective targeted drugs are limited.

### 5.1. MET

MET is a receptor for hepatocyte growth factor that has been implicated in growth, invasion, and metastasis of cancer cells. Dysregulation of MET enhances the malignant properties of NSCLC and defines a subset of patients that might potentially benefit from anti-MET targeted therapy [35]. In fact, for NSCLC patients with MET exon 14 skipping (METex14) mutation, treatment with an MET inhibitor has been demonstrated to be associated with an improvement in overall survival [36]. In NSCLC, METex14 occurred in approximately 0.6% (221/38028) of the patients with advanced disease [37]. The incidence of MET exon 14 mutation, which included METex14 and amplification, in NSCLCs is approximately 3%, and the mutation is more likely to occur in older female nonsmokers; both METex14 (HR = 2.156, *P*=0.026) and MET amplification (HR = 3.444, *P*=0.007) have been correlated with poor patient prognoses in NSCLC in multivariate analyses [38, 39]. Compared with common NSCLC, mutation rates of MET are high in PSC. In the Western population, the incidence of METex14 was 12.0% (15/125) in the cohort by Schrock et al. and 22.2% (8/36) in the cohort reported by Liu et al. [29, 40]. In a Chinese population (*n* = 687), the incidence of METex14 mutation in PSC was 31.8%, compared to that of 2.6% in adenocarcinoma, 4.8% in adenosquamous carcinoma, and 2.62% in all NSCLCs [39]. The incidences of MET mutation and METex14 mutation in our cohort are 19.6% (11/56) and 10.7% (6/56), respectively [34]. The results implicated that mutational incidents of MET, specifically METex14, are frequent events in PSC and that MET inhibition might benefit this specific subgroup of patients [40].

#### 5.1.1. Savolitinib

Savolitinib is an oral, selective MET tyrosine kinase inhibitor developed for the treatment of metastatic NSCLC, papillary and clear cell renal cell carcinoma, gastric cancer, and colorectal cancer [41]. The first case of response to savolitinib in a PSC patient with METex14 mutation was reported by Han et al., in which significant clinical benefit was achieved with savolitinib 600 mg orally, once-daily treatment in a 75-year-old male patient with a heavy smoking history [42]. The patient was one of the participants in NCT02897479, a single-arm, multicenter, open-label, phase 2 study that covered 32 hospitals in China, and the largest trial of MET inhibitors in PSC. In NCT02897479, savolitinib monotherapy was tested in 70 locally advanced or metastatic NSCLC patients with METex14 mutation; the ORR by the independent review committee was 49.2% (30/61) in all evaluable patients, and there was not any complete response [43]. In the 25 PSC patients, the achieved ORR was 40.0% (10/25) and 8 patients had stable disease [43]. In the 70 treated patients, only one death due to tumor lysis syndrome was reported in a PSC patient (1.4%, 1/70) and the incidence of grade 3 or higher treatment-related adverse effects was 45.7% (32/70) [43]. Based on this, savolitinib was approved in June 2021 by the National Medical Products Administration to treat metastatic NSCLC patients with METex14 mutations who have progressed after platinum-based chemotherapy or are unable to tolerate it [41, 44]. In August 2021, a multicenter, open-label, phase III confirmatory clinical trial to evaluate the efficacy, safety, and tolerability of savolitinib in treating locally advanced or metastatic NSCLC patients with METex14 mutations was initiated; the study was planned to enroll 163 participants, and the result may offer further support for the treatment of PSC using savolitinib [45].

#### 5.1.2. Crizotinib

Crizotinib is an oral small-molecule, multikinase inhibitor of ALK, ROS1, RON, and MET and has been approved for the treatment of advanced NSCLC patients harboring ALK or ROS1 rearrangements; it acts as a type Ia MET tyrosine kinase inhibitor and competes for the ATP-binding site of the RTK, thereby preventing activation of downstream signaling pathways [46]. Response to crizotinib targeting METex14 in NSCLC was initially reported by Waqar et al. in a 71-year-old male patient with METex14; crizotinib 250 mg orally twice daily was prescribed to the patient; computed tomography scans 6 weeks after treatment initiation showed a decrease in lesion size, and the response continued for at least 6 months [47]. In another report, Lee et al. reported the efficacy of crizotinib in a 61-year-old never-smoker PSC patient with METex14 mutations; after failing the combination of radiation, carboplatin, paclitaxel, and bevacizumab treatment in the ECOG5508 trial (NCT01107626), he was given crizotinib and experienced symptomatic improvements in activity level and pain [48]. To date, no prospective clinical study has been conducted to investigate the effect of crizotinib on PSC yet [9, 10].

#### 5.1.3. Capmatinib and Tepotinib

Targeting MET mutation with capmatinib or tepotinib has been demonstrated to be effective and safe in MET-dysregulated advanced NSCLC, with rapid and durable responses observed across the cohorts [49, 50]. Based on the results, approvals of capmatinib and tepotinib for patients with metastatic NSCLC harboring METex14 were granted in May 2020 and September 2019 by the US Food and Drug Administration [51]. There were 3 PSC patients included in the VISION study, but the responses of them were unknown [50]. Otherwise, no relevant case or cohort study on PSC has been reported yet.

### 5.2. EGFR and KRAS

Epidermal growth factor receptor-tyrosine kinase inhibitor (EGFR-TKI) treatments are recommended for NSCLC patients with two most common sensitizing mutations (exon 19 E746-A750 deletion and exon 21 L858R) [11]. The two mutations are oncogenic and activate the EGFR signaling pathway in the absence of ligand, promoting downstream prosurvival and antiapoptotic signals of tumor cells [52]. KRAS is a G-protein with intrinsic GTPase activity, and mutations of KRAS in PSCs were associated with poor survival [11, 53, 54]. Patients with KRAS mutations have been associated with reduced responsiveness to EGFR-TKI therapy [11].

EGFR-TKIs are potential therapeutic drugs for PSC, but their clinical effects have not been validated. In a retrospective analysis of 22 PSC cases, EGFR protein overexpression was observed in all the cases; KRAS mutation was detected in 38.1% (8/21) of the patients, while no EGFR mutation was detected; the researcher thus concluded that most patients with PSC are not likely to benefit from EGFR-targeting therapies due to the rare incidence of increased EGFR gene copy number, the lack of EGFR mutation, and the high rate of KRAS mutation [55]. However, recent studies led to disparate conclusions. For example, in our research, the frequency of common EGFR mutations was 16.0% (9/56 cases), which indicated a higher mutation rate in the Chinese population than that in the Western populations (8.8%, 11/125 cases) [29, 34]. Based on the omics results, PSC could be theoretically treated with EGFR-TKIs. Several EGFR-TKIs, including gefitinib, erlotinib, afatinib, and osimertinib, have been developed and approved for first-line treatment of advanced NSCLC harboring EGFR-activating mutations [11]. However, the effect of them in PSC has not been validated in clinical trials yet.

#### 5.2.1. EGFR-TKIs

There are only a few cases that reported the effect of EGFR-TKIs in PSC patients. In a report by Zou et al., a 73-year-old female PSC patient was successfully treated with erlotinib after failing chemoradiotherapy and remained progression-free for 6 months after EGFR-TKI treatment [56]. In a cohort by Lin et al., 5 patients received gefitinib for palliative treatment and 3 patients maintained stable disease for 2, 3, and 5 months, respectively [2]. While the effect of monotherapy seems limited, combination therapy may confer a better antitumor effect. In a report by Wang et al., the combination of crizotinib and gefitinib led to PR in a 74-year-old female PSC patient with concurrent EGFR-activating mutation in exon 21 L858R and MET amplification [57]. In another report by He et al., afatinib combined with crizotinib led to partial response and was well tolerated in a PSC patient with a double-rare L747S, G719S EGFR mutation and a secondary MET amplification [58]. However, EGFR-TKIs have also been reported to promote the transformation of lung cancer into unfavorable pathologic types [59]. In summary, the effect of EGFR-TKIs remains to be verified in clinical studies.

### 5.3. ALK

ALK is a receptor tyrosine kinase that can be rearranged in NSCLC, resulting in dysregulated signaling through the ALK kinase domain [60]. First-line therapies for ALK rearrangement positive NSCLC include alectinib, brigatinib, ceritinib, crizotinib, and lorlatinib [11]. There are only limited reports of PSC patients treated with anaplastic lymphoma kinase-tyrosine kinase inhibitors (ALK-TKIs).

#### 5.3.1. ALK-TKIs

Antonio et al. reported a 46-year-old female nonsmoker case with both ALK rearrangement and high levels of PD-L1 expression receiving ALK-targeting therapy; being intolerable to second-line crizotinib, the patient underwent 5 cycles of ceritinib treatment and achieved partial remission [61]. Miao et al. reported an ALK-positive PSC patient, who experienced tumor shrinkage 3 months after the combination therapy of crizotinib and radiation [62]. Similarly, no clinical trial has been conducted to investigate the effect of ALK-TKIs in PSC yet [9, 10].

### 5.4. Anlotinib

Anlotinib is a potent multityrosine kinase inhibitor that could suppress angiogenesis and inhibit the activation of VEGFR2, PDGFR*β*, and FGFR1 as well as their common downstream ERK signaling; in the phase III study ALTER 0303, anlotinib significantly increased OS and PFS in patients with advanced NSCLC after at least two lines of treatment [63, 64]. A case of anlotinib was reported by Li et al. in a 77-year-old male PSC patient; after disease progression following first-line chemotherapy, he was prescribed anlotinib combined with second-line chemotherapy (dacarbazine and cis-platinum) for six cycles, and the response reached complete remission [65]. In addition, a PSC patient receiving anlotinib combined with nivolumab was reported to achieve partial remission 8 weeks after initial treatment [66].

### 5.5. Apatinib

Apatinib is a vascular endothelial growth factor receptor-2 tyrosine kinase inhibitor and an antiangiogenic drug [67, 68]. Blood-vessel invasion has been presented as a major feature in most (90.7%, 68/75) of the PSCs [69] and was related to a poor prognosis [33]. Li et al. reported the efficacy of apatinib in a 75-year-old stage IV PSC patient, who complained of coughing and hemoptysis for over one month and presented chest stuffiness and shortness of breath that required continuous oxygen inhalation [70]. Being capable of only limited self-care, the patient was precluded from chemotherapy; CD31 and CD34 immunostaining in the biopsy sample showed abundant microvessels in the tumor, indicating active tumor angiogenesis; thus, the patient was prescribed apatinib (250 mg) orally once per day; the prescription relieved chest stiffness and eradicated hemoptysis 5 days after treatment [70]. In another report, Kong et al. reported a series of inoperable PSC cases that received albumin-bound paclitaxel and carboplatin plus apatinib as first-line therapies [71]. The evaluation of response was partial remission in two patients and stable disease in one, on the 6-month follow-up, whereas one of the patients had progressive disease on the 7-month follow-up [71]. The results indicated that antiangiogenesis therapy may be an alternative therapy for patients with advanced PSC.

### 5.6. Other Treatments

ROS1 mutation is one of the targets of crizotinib. For a spindle cell carcinoma patient with a ROS1 fusion mutation, clinical benefit from crizotinib was reported; partial remission was achieved one month after crizotinib initiation in a 72-year-old Chinese male patient [72]. In addition, Manzotti et al. reported that the transcriptional activation of an epithelial mesenchymal transition program drives the phylogeny and that dasatinib, a broad-range tyrosine kinase inhibitor approved in the treatment of NSCLC, might be a new therapeutic option [73].

## 6. Immunotherapy

Immune checkpoints are a category of costimulating surface proteins that transmit inhibitory signals on T cells [74]. Among these proteins, programmed cell death protein 1 (PD-1)/programmed cell death 1 ligand 1 (PD-L1) were the most studied ones, and the blocking antibodies of them have been approved to treat a wide variety of cancers [75, 76]. Up to now, anti-PD-1/PD-L1 therapies have been recommended as first-line therapies for advanced or metastatic NSCLC with positive PD-L1 expressions and immunohistochemistry-detected PD-L1 expression is the only approved biomarker in the National Comprehensive Cancer Network guideline to select patients who are candidates for PD-1/PD-L1 inhibitors [11].

### 6.1. Expression of PD-L1 and Tumor Mutation Burden in PSC

Generally, the expression of PD-L1 in PSC is high and the positive rate of PD-L1 in PSC is higher than common NSCLC. In a report from two large retrospective lung cancer cohorts, high expression levels of PD-L1 in PSC were detected using automated quantitative immunofluorescence, with an approximately 40% higher PD-L1 level in PSC than in conventional NSCLC (automated quantitative fluorescence analysis score of 138 and 190, respectively, *P* < 0.01) [77]. In 13 patients diagnosed with PSC, 9 (69.2%) were positive for PD-L1 (cutoff unknown) and the positive rate was higher than that in conventional NSCLC (27.4%, 122/445) [77]. The positive rate for PD-L1 (≥1%) in PSC in a pooled analysis was 89.4% (59/66), while high expression levels of PD-L1 (≥50%) were observed in 74.2% of the patients (49/66) [78]. In another retrospective study by Vieira et al., positive PD-L1 (≥5%) staining was observed in tumor cells in 53.3% (40/75) of the cases by immunohistochemistry and the levels of PD-L1 were also higher in PSC than those in adenocarcinoma (25.0%, 5/20), squamous carcinoma (15.8%, 3/19), or large cell carcinoma (20.0%, 3/15) [69]. When the cutoff value of positive PD-L1 was set at 10%, the positive rate for PD-L1 in PSC turned to 25.6% (11/43), while the median value was 4% [79].

PD-L1 positive correlates with mutational load and is an unfavorable prognostic factor for PSC. In the study by Lococo et al., all 11 PSC patients that scored positive for PD-L1 staining presented at least one mutation in the panel of genes analyzed [79]. PD-L1 was strongly associated with the presence of KRAS mutation (*P*=0.031), and 73% of PD-L1-positive cases have concomitant KRAS mutations [79]. Consistent with this, PD-L1 expression was not only associated with aggressive pathological features of PSC, such as N2 involvement, and metastasis but also slightly correlated with reduced overall and disease-free survival probabilities (*P*=0.069 and 0.015, respectively) in PSC patients [79]. In our cohort, PD-L1 expression correlated with the expression of CD47, a ubiquitously expressed transmembrane glycoprotein that has been proved to suppress phagocytosis from macrophages and help tumor cells evade from the immune system [80]. Coexpression of PD-L1 and CD47 correlated with a poorer prognosis and may serve as a predictive biomarker for combined dual-targeting immunotherapy in PSC patients [80].

The mutational burden of PSC is high. The median tumor mutation burden (TMB) was 8.6 mutations/Mb in Chinese PSC patients, and more than 60% of the patients (65.5%, 38/58) were characterized as microsatellite instability-high, PD-L1-positive, or high-TMB, which indicated possible survival benefits from ICIs [30]. There was no difference in TMB between carcinomatous component and sarcomatous component [81, 82]. However, a higher median TMB was observed in PSC compared to lung adenocarcinomas (10.1 versus 4.8 mutations/Mb, *P*=0.003) [82]. In our research, the TMB of PSC was 6.9 mutations/Mb on average, slightly lower than that of lung adenocarcinoma (7.03 mutations/Mb, *P*=0.037) and lung squamous carcinoma (8.03 mutations/Mb, *P*=2.545 × 10^−5^), and higher than that of sarcoma (1.45 mutations/Mb, *P*=3.781 × 10^−13^) [34]. Moreover, it has a higher leukocyte fraction than other TCGA tumor types, which suggested a T-cell inflamed microenvironment of PSC and the suitability for immunotherapy [34]. Based on these results, efficacy of ICIs was explored in PSC.

### 6.2. Efficacy of ICIs in PSC

Preliminary antitumor efficacy of ICIs has been observed in PSC in retrospective studies, and the effect of anti-PD-1 immunotherapy seems better in PD-L1-positive patients compared with PD-L1-negative ones. Domblides et al. conducted a retrospective analysis that included 37 PSC patients receiving anti-PD-1 immunotherapy as a second-line treatment or beyond following platinum-based chemotherapy [83]. Nivolumab was the main drug for immunotherapy, and after a median cycle of 10, overall response was achieved in 40.5% (15/37) of the patients, while disease control was achieved in 64.8% (24/37) of the patients [83]. Among 19 patients with available samples for PD-L1 detection, only one was PD-L1 negative; the ORR was 58.8% in PD-L1+ patients and 0% in the single PD-L1− patient; a trend toward better OS was also observed in PD-L1+ patients, but there was no statistical significance [83]. In another retrospective study conducted by Manglaviti et al., 19 patients with metastatic sarcomatoid carcinoma were treated with ICIs; the achieved ORR was 31.6% (6/19), DCR was 36.8% (7/19), median PFS was 2.3 months, and median OS was 3.5 months [84]. In a pooled analysis that included the abovementioned 2 studies, Babacan et al. found that PD-L1 expression is significantly associated with favorable tumor responses and PFS after checkpoint inhibitor immunotherapy; among patients with available tissue for PD-L1 detection, partial or complete response was achieved in 70.2% (33/47) of the patients with high PD-L1 expressions (≥50%), compared with 50% (5/10) of patients with a PD-L1 of 1%–49% and 28.6% (2/7) of patients with PD-L1 <1% (*P*=0.026); PFS was longer among patients with PD-L1≥1%, compared with those with PD-L1 <1% (14.4 months versus 2.7 months, *P*=0.04) [78]. Thus, immunotherapy produces better outcomes in PD-L1-positive PSC patients than in PD-L1-negative ones, whereas immunotherapy may still be efficacious in PD-L1-negative patients, which might be due to the interaction between the immune system and tumor cells [78].

Nivolumab is an anti-PD-1 antibody approved for the treatment of NSCLC [85]. Specifically, there has been a special report showing the antitumor effect of nivolumab in an METex14-mutated PSC patient [86]. A French female patient with METex14-mutated stage IV PSC was given nivolumab as a second-line therapy after failing first-line chemotherapy; surprisingly, although there is no predictive factor of response to immune checkpoint inhibitors (ICIs), rapid complete response was achieved and maintained for 25 months and she was recurrence-free 16 months after nivolumab initiation [86]. This case indicated that ICIs may be a possible choice for METex14-mutated patients.

Currently, there have been no available results of prospective studies on PSC. However, there are some ongoing trials [9, 10]. Durvalumab is a selective human immunoglobulin G1 monoclonal antibody against PD-L1, and tremelimumab is a selective human immunoglobulin G2 monoclonal antibody binding to cytotoxic T-lymphocyte-associated antigen-4; the combination of durvalumab and tremelimumab has shown preliminary antitumor efficacy in locally advanced or metastatic NSCLC, with a manageable tolerability [87]. In an open-label, multicenter, single-arm phase II study (NCT03022500), the effect of durvalumab plus tremelimumab was tested in metastatic/relapsed PSC; the study was initiated in June 2020, and its primary endpoint has been met [88]. In 15 evaluable patients, the ORR was 26.7% (4/15) and the DCR was 60.0% (9/15); median PFS and OS were 5.9 months and 15.4 months, respectively [88]. In addition, effects of PD-1 inhibitors, toripalimab and camrelizumab, are also being tested in ongoing trials and the results are worth expecting [9, 10].

### 6.3. ICIs in Combination with Apatinib

Recently, apatinib in combination with ICIs has shown a promising effect in NSCLC treatment. Preclinical and clinical evidence has shown that low-dose apatinib could improve the effect of anti-PD-1/PD-L1 immunotherapy. In a murine Lewis lung carcinoma model, low-dose apatinib alleviated hypoxia, increased lymphocyte infiltration, and reduced PD-L1 expression on the tumor, remodeling the immunosuppressive tumor microenvironment to a more permissive one for antitumor immunity; combining low-dose apatinib with anti-PD-L1 significantly retarded tumor growth, reduced the number of metastases, and prolonged survival of mice [89]. In a proof-of-concept clinical trial, apatinib plus camrelizumab resulted in an ORR of 27.6% (29/105) in chemotherapy-pretreated and immunotherapy-naive patients with nonsquamous NSCLC [90]. The results have implicated the promising prospect of apatinib plus anti-PD-1/PD-L1 immunotherapy as a novel treatment modality for PSC, although there have been no clinical trials or case reports testifying this at present.

## 7. Conclusion

PSC is a subgroup of NSCLC with a poor prognosis that may be attributed to a considerable extent to the resistance to chemotherapy as well as radiotherapy. However, the prevalence of mutations in this type of tumor laid the foundation for novel systemic therapies such as targeted therapy and immunotherapy. Furthermore, understanding of the mechanisms underlying the pathogenesis of this disease may contribute to novel therapeutic strategies.

Similar to other types of NSCLC, surgery is the optimal choice for resectable PSC, but most patients are in an advanced stage at diagnosis [6]. Radiotherapy and chemotherapy have controversial effects, and the effects of them need to be confirmed in large sample studies in different settings. Meanwhile, the progress made in targeted therapy and immunotherapy has shed light on the treatment of advanced disease. Savolitinib is the only approved drug that was tested in a prospective clinical trial and has been approved in treating metastatic patients with METex14 mutations. Other targeted drugs, such as crizotinib, ceritinib, gefitinib, afatinib, anlotinib, and apatinib, have been reported to confer antitumor effects, but the effects have not been verified in prospective clinical trials yet. The expression of PD-L1 in PSC is high, and immunotherapies such as nivolumab and durvalumab plus tremelimumab could lead to tumor responses.

**Figure 2:**
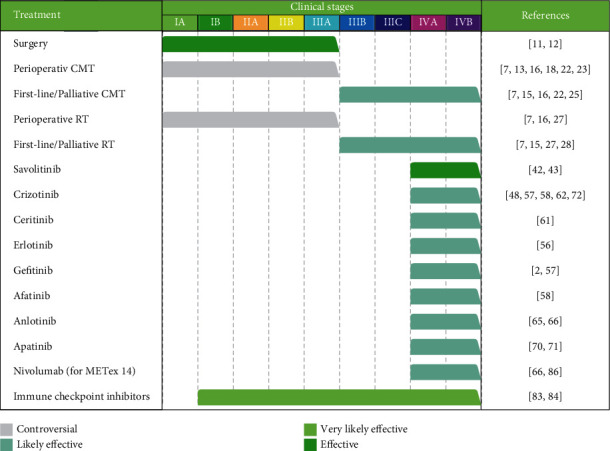
Summary of current therapeutic regimens for pulmonary sarcomatoid carcinomas. Clinical stages were based on the eighth American Joint Committee on Cancer lung cancer stage classification (PMID: 27780786). CMT, chemotherapy; RT, radiotherapy; METex14, MET exon 14 skipping.

To date, there have been few prospective studies on PSC, perhaps because of the rarity of the tumor; most of the related literature was case reports or cohort studies. The treatment regimens for patients with PSC are shown in Figure 2, which are summarized based on research articles discussed above. Further research is needed to confirm the effects of various systemic therapies, and multimodality treatments are required to maximize the benefit for patients with advanced disease.

## Data Availability

No data were used to support this study.
